# Buffalo Medical and Surgical Association

**Published:** 1885-05

**Authors:** 


### Society Reports.

### Buffalo Medical and Surgical Association.

*Meeting of March 3, 1885.*

The President, Dr. F. W. Bartlett, in the Chair; Dr. Frank H. Potter, Secretary.

Dr. Gager, being present, was, on motion, invited to take part in the proceedings of the meeting as the guest of the association.

*Extra-uterine Pregnancy.*—Dr. Matthew D. Mann read a paper on this subject, reporting four cases, with the treatment and results. He prefaced these cases with a brief *résumé* of the symptoms leading to the diagnosis of this condition. These were, of course, the symptoms of an ordinary pregnancy, including the mammary and gastric signs. Particular importance must be given to the changes in the mammæ, as in the majority of cases they were among the most reliable symptoms, if the pregnancy had advanced far enough. Besides these, as was mentioned by other writers, there were irregular gushes of blood, accompanied by portions of membranes, or, as he himself had seen, a complete uterine decidua, much hypertrophied and thickened. With the microscope we could distinguish this from the menstrual decidua. Then there was pain, either fixed and grinding in the iliac fossæ, or of a sudden, paroxysmal character, of the severest kind and accompanied by symptoms of shock. Quite rarely there were symptoms of abortion without the expulsion of a fœtus. Again, in a small proportion of cases, there were no premonitory symptoms, the first indication of anything wrong being shock, collapse, and sudden death. The physical signs were, commonly: 1. Increased size of the uterus, but not in proportion to the supposed stage of the pregnancy. This increase in size was thought by some to be proportionate to the nearness of the sac to the uterus, the increase being greater in the tubal than in the abdominal variety. 2. Evidence of the emptiness of the uterus by the passage of the sound or a finger. 3. The presence on one side of or behind the uterus of a cystic tumor, generally smooth in outline, somewhat tender, and obscurely fluctuating. With these signs and symptoms clearly in mind, a diagnosis, in the majority of cases, could be made with certainty. And moreover, when the diagnosis could be made early, treatment could be applied with a reasonable assurance of success. He then reported in detail three cases of extra-uterine pregnancy which had been successfully treated by the application of the electric current. The diagnosis in each case was made early, in accordance with the above-mentioned principles. The Faradaic current was used. This was passed through the tumor daily, and was continued for a week or more, according to indications. Each patient made a satisfactory and complete recovery.

The fourth case, not having had so favorable a termination, was reported more at length. It occurred in the practice of Dr. Diehl, of Buffalo. The patient was forty-two years of age. She had had one child eighteen years before, but no miscarriages. Dr. Diehl first saw her on October 7, 1883, on account of excessive bleeding from the uterus. This was controlled after a time with ergot. On January 4, 1884, he was compelled to use a catheter on account of retention of urine. In the meantime she had menstruated, but at irregular intervals, and had occasionally complained of severe pain in the lower extremities and in the bowels. On April 1st a tumor was discovered in the left lower portion ot the abdomen. Dr. Mann saw her on the 10th, when the tumor was found extending nearly to the umbilicus and situated entirely to the left of the median line. Fœtal life was easily detected. Extra-uterine pregnancy was considered probable, but, as she insisted that she had carried her first child in the same situation, it was doubtful whether it might not be a pregnancy in the left horn of a uterus unicornis or bicornis. For this reason the uterine cavity was not explored. From this time until the 1st of August she continued to do well, only suffering occasional pain, not severe. On the latter date, as the end of pregnancy was so near, and as but little harm would result from a premature labor, it was determined to examine the uterine cavity. This was found to be enlarged but empty. Pregnancy was now so far advanced that it seemed best to await the false labor, which usually occurs in these cases, and the death of the child, before operating. Fœtal life ceased to manifest itself about the latter part of August, the cessation being preceded by pains simulating those of labor. She continued to do very well, however, no urgency occurring in the symptoms until the night of the 24th of September, when, as she was straining at stool, she was taken with severe pain, accompanied with the sensation of something giving way. When Dr. Diehl saw her, early in the morning, she was in a state of collapse. Dr. Mann was hastily summoned and operated at 10 A. M., assisted by Dr. Diehl, Dr. Rochester, Dr. Phelps, and Dr. Bartow. Before the operation the patient was conscious and hopeful. The peritoneal cavity was found filled with a thin purulent fluid, which was first removed. The fœtus was then removed, the cavity washed out thoroughly with antiseptic solutions, and the wound closed. The patient failed rapidly during the operation, and died soon after it was completed. The autopsy revealed a large sac external to the uterus and attached to it and to the rectum. The appearances indicated that the sac was the enlarged and hypertrophied oviduct of the left side. The uterus contained a decidua somewhat thicker than normal. From the study of these cases and those reported by other writers, Dr. Mann then formulated the following considerations for the treatment of extra-uterine pregnancy:

1. If the case was not seen until the alarming symptoms attending rupture of the sac have been developed, laparotomy must be performed without delay. Mr. Lawson Tait's four successes out of five cases must encourage us to follow his example.

2. If the pregnancy was discovered before the fourth month, the indication was to destroy the fœtus and trust to nature for its absorption. All other plans for accomplishing this purpose must be laid aside in view of the safe and easy method offered in the application of the electric current. This might be in the form of the interrupted Galvanic or the Faradaic current, the latter being considered the most reliable. It should be used daily, as strong as could be borne, and continued until there was evidence of the death of the fœtus.

3. Opinions differed as to the proper plan to pursue after the fourth month. If the fœtus was alive, it should be destroyed by electricity, no matter at what stage the pregnancy might be. If it was dead and in the earlier months, nothing should be done until some clear indication for operating occurred. In the latter months, after its death, it was better to wait a few weeks before operating. By this plan the danger from hemorrhage would be lessened, as well as all the difficulties surrounding the removal of the placenta. On the other hand, if the fœtus was left to be absorbed by nature, there was great danger of suppuration and septicæmia.

In the discussion, Dr. Wetmore called attention to the fact that we hear of more cases of extra-uterine pregnancy to-day than formerly, probably because of our more accurate diagnosis. He had had one case out of 1,400 deliveries. In that case he used the Galvanic current of the strength of twenty cells with success. He was of the opinion that the constant current was the best.

Dr. Cronyn reported a case of double uterus, which, at first, he thought to be an extra-uterine pregnancy, but in a normal labor the woman was delivered of a living child. While electricity was the proper treatment in some selected cases, in others it would be better to perform laparotomy, and in still others nature would succeed in bringing about a successful termination without the interference of the physician.

Dr. Hartwig thought more cases of extra-uterine pregnancy occurred than were reported, perhaps because they were not recognized. He called attention to the fact that hæmatocele sometimes resulted from these cases. He had once performed laparotomy for suspected extra-uterine pregnancy, but had failed to find the fœtus.

Dr. Stockton reported a case of suspected abortion which the autopsy revealed to have been an extra-uterine pregnancy. Death resulted from hemorrhage one and a half hours from the first symptom. Even if there had been no suspicion of abortion, death followed so soon after the first symptom that it is difficult to see how any interference on the part of the physician could have been successful.

Dr. Moody inquired as to the effect of abortifacients—as ergot—upon extra-uterine pregnancy.

Dr. W. W. Potter said that danger existed from the outset of an extra-uterine pregnancy, and was immediate and remote, and therefore the treatment could be considered under two heads, remedial and operative. If the case was under observation early, the operation should be deferred until there was some plain indication for its performance. He considered it was now settled that the proper time to interfere by laparotomy in these cases was immediately upon the death of the fœtus, whether caused by electricity or by nature.

He also mentioned the possibility of a normal pregnancy existing at the same time, and wished to impress the necessity of having this occurrence always in mind.

Dr. Mann, in closing the discussion and replying to Dr. Moody, said that abortifacients could have no effect upon an extra-uterine fœtus, for those remedies acted by stimulating the muscular fibres of the uterus to contract, not by any direct action upon the contents of the womb. In extra-uterine pregnancy motion was observed earlier than in normal pregnancy, and this was a valuable aid in diagnosis. It was his opinion that these cases occurred more frequently than was generally expected. Hæmatocele could result from an extra-uterine pregnancy.

The prevailing diseases reported were diphtheria, croup, scarlet fever, measles and pneumonia.

Dr. Brecht presented a specimen of lithic acid from a child two weeks old. The child had had convulsions continually since its birth.

The committee upon midwifery reported a corrected bill regulating that practice by other than regular physicians in Erie county.

After some discussion the President was instructed by motion to ask the President of the Erie County Medical Society to call a special meeting for the consideration of this bill.

The association then adjourned.

